# Extreme weather and climate events in China under changing climate

**DOI:** 10.1093/nsr/nwaa069

**Published:** 2020-04-17

**Authors:** Weijie Zhao

**Affiliations:** An NSR news editor based in Beijing

## Abstract

In the summer of 2003, a heat wave swept Europe and caused more than 70 000 additional fatalities [J.-M. Robine *et al.*, *C. R. Biologies***331** (2008)]. Global warming and climate change is no longer a prophecy to be fulfilled, as strong heat waves and typhoons, as well as severe rainfalls, are becoming more severe. Extreme weather and climate events in the world, especially over Europe and North America, are widely studied and frequently reported in the media. These events may be related to the ongoing climate change. In this NSR forum, active researchers specialized in this field gather to discuss the climate and weather changes in China. They present the current changes, identify knowledge gaps, discuss the research difficulties, and propose ways forward to better serve the society with climate science.

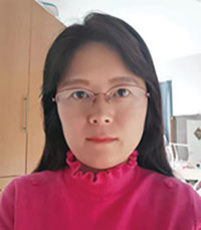

**Ying Sun**

Professor at the National Climate Center of the China Meteorological Administration, China

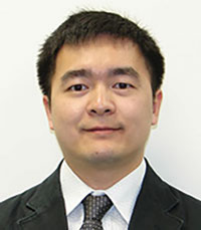

**Qiuhong Tang**

Professor at the Institute of Geographic Sciences and Natural Resources Research of the Chinese Academy of Sciences, China

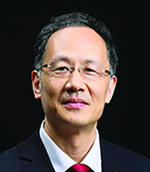

**Zhongwei Yan**

Professor at the Institute of Atmospheric Physics of the Chinese Academy of Sciences, China

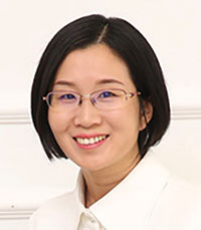

**Jing Yang**

Professor at the Academy of Disaster Reduction and Emergency Management, faculty of Geographic Science of Beijing Normal University, China

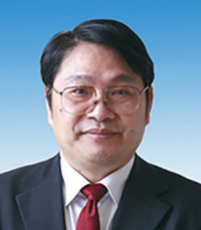

**Panmao Zhai**

Professor at the Chinese Academy of Meteorological Sciences of the China Meteorological Administration, and the current Co-Chair of the IPCC Working Group I, China

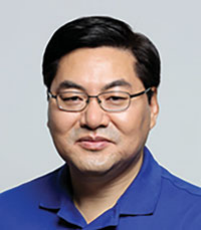

**Tianjun Zhou**

Professor at the Institute of Atmospheric Physics of the Chinese Academy of Sciences, China

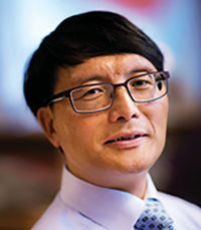

**Deliang Chen (Chair)**

Professor at the University of Gothenburg, NSR Editorial Board member, Sweden

## CHANGES IN CHINA

**Chen:** Can we first take a look at how extreme weather and climate in China has been changing against the global warming background?

**Yan:** Over the past century, global warming is a fact and China has been warming faster than the world average. The world average temperature rise within the past century was less than 1°C, but for China, it was 1.3–1.6°C according to the updated studies.

Greater temperature rise leads to stronger impacts. In China, heat waves are increasing; severe rainfalls are increasing; light rainfalls are decreasing. The increase of extreme heat waves and rainfalls means that the meteorological factors for both drought and flood are also increasing. But of course, whether the meteorological factors will lead to disastrous droughts and floods also depends on how we act to adapt to the climate change.

In 2013, a 2-month heat wave shrouded East China. Researchers estimated the total economic loss in Nanjing to be around 3.4% of the city's annual GDP. The economic loss calculated here includes both direct and indirect losses. The modern society is a highly connected complex system. The impact of a local disaster can spread to the whole area and diverse industries through the industrial chains and social networks, causing cascading indirect losses. Moreover, the impacts of heat waves

In China, heat waves are increasing; severe rainfalls are increasing; light rainfalls are decreasing.

—Zhongwei Yan

are not limited to economic loss, but also include damage to human health, causing more diseases and deaths of certain groups of people.

**Yang:** The increased connectivity, complexity and population density of the modern society lead to increasing vulnerability of the system, which means that extreme events of a similar level could cause damages that are more serious than before. On the other hand, it also means that when we study the extreme events, we need to pay more attention to detailed changes in time/space, but not only the changes of basic indices such as frequency and intensity.

As an example, for extreme high temperature events, the influential area, the duration and the amplitude/frequency of variation over time are all important factors. In China, heat waves are becoming more frequent and affecting larger areas. The impact is not limited to East China, but also has extended to North China and even Northeast China in recent years. And for human health, large temperature fluctuations should also be considered, because they are usually more harmful than stable lasting high temperatures.

The same is true for typhoons. Changes in Western Pacific typhoons under global warming are less well studied compared with Atlantic hurricanes. But we can see that for the Pacific typhoons affecting China, the genesis number has dropped over some basins in recent decades but the intensity has increased. As the overall sea surface temperature increasing, the genesis positions and tracks of typhoons are also moving northward. More typhoons are hitting Japan and the Bohai Bay of China. Some strong ones can even reach Northeast China.

**Zhou:** Some Chinese scientists claim that the Earth has experienced dramatic climate changes over the billions of years of Earth's history, so that the current and coming climate change of several degrees is not a big deal. This is right. But the problem is that we humans are living on this planet right now. Several degrees’ climate change can bring disasters to human society. When we talk about climate change issues, we should clarify the time scale. We are not talking about the Earth's ancient history, but the climate changes since the industrial revolution, which are to a large extent caused by human activities and will further affect human society.

## ATTRIBUTION: CAUSES FOR THE CHANGES

**Chen:** Attribution is a major research topic. Professor Sun did many works in this field. Would you like to share your views?

**Sun:** Attribution aims at answering two kinds of questions: What are the causes of the observed changes in climate such as warming? Have human activities altered the probability and/or magnitude of a particular type of extreme event like the one that has just occurred that caused large loss and damage?

Attribution relies on both observational data and climate models. We first assume that the models are trustworthy enough to well describe the relevant physical processes, and then compare model simulated climate responses to different forcings with the observations to determine if observed changes are due to particular forcings. We also compare model simulations with or without anthropogenic forcing to infer if anthropogenic activities have changed the odd or magnitude of particular type of extreme events.

Our work, as well as those conducted by other scientists, provide us the confidence to say that observed warming in China is predominately due to the emission of greenhouse gases. Additionally, the increase of warm temperature extremes and the decrease of cold extremes in the past decades are very likely to be attributed to human activities dominated by greenhouse gas emissions. The human influence also increases the probability of extreme high temperature events.

Attribution of other events is more complex. For instance, it has long been difficult for the models to reproduce the observed changes in precipitation. Models generally show poor performance in simulating heavy precipitation. Change in precipitation is still small relative to precipitation variability, making it hard to attribute changes in precipitation, especially at regional scale. With global warming, the anthropogenic forcing continues to increase and we are beginning to see influence of human activity on heavy precipitation in some regions.

Drought is even more complex. It generally lasts for a long time and involves more factors and indicators, so that the current attribution works are limited. Currently, attribution works within China are focused on the above mentioned fields. Works on typhoon and other events are relatively few.

## MONSOON AND PRECIPITATION

**Zhai:** China is greatly influenced by the monsoon system, but the attribution of monsoon changes is still not clear. Many believe that changes in the monsoon system are basically natural and cannot be attributed to human activities. It is a complex system involving atmosphere–land–ocean interactions, atmospheric circulation, aerosol influences and more.

Extreme precipitation is strongly influenced by monsoon activity. If we cannot clarify human activity's influence on East Asian monsoons, it would be impossible to understand human influence on precipitation. Currently, different models give similar simulation results: precipitation in North China and southern China would both increase under the global warming

background. But observation shows that East Asian monsoons are generally weakening, with precipitation in southern China increasing and precipitation in North China decreasing.

If we cannot solve the attribution problems of changes in monsoon and precipitation, we will be unable to connect global warming with extreme events, or to make projections. ‘Climate change’ would remain a descriptive story but could not explain real-world weather and climate in our region.

It is complex and there are differences between East and South Asian monsoons.

—Ying Sun

**Sun:** I did some work on monsoons. It is complex and there are differences between East and South Asian monsoons. With global warming, the South Asian summer monsoon precipitation is substantially influenced by thermodynamic factors, whereas summer monsoon precipitation in East Asia is more closely associated with dynamic factors such as the enhanced low-level southerly winds in southern China. My personal view is that, in the coming decades, the monsoon system will be co-influenced by natural and human factors; but as the human influence increases, when global warming exceeds a certain level, the influence of human activity would become more obvious and significantly affect the monsoon system.

**Yan:** At a recent conference of the Climate Science for Service Partnership China (CSSP-China), we also discussed the issues of monsoons and precipitation. We thought that a major direction for monsoon attribution would be to attribute the abnormal behaviors of the atmospheric circulation. In many state-of-the-art climate models, the summer monsoon does not reach North China, so we cannot see the monsoon's contribution to the precipitation of North China. If we can identify causes for circulation changes, we may be able to better attribute monsoon and precipitation. Finally, by increasing models’ spatial resolution and through downscaling, we may improve our chances of successfully attributing precipitation change and reducing uncertainty in future projections of precipitation.

## DROUGHTS AND FLOODS

**Chen:** Droughts and floods are more complex. They are based on precipitation but also involve the whole water cycle and many hydrological factors, including those on the underlying surface. We have a hydrological expert, Professor Tang, here to give some insight.

**Tang:** Defining a suitable index for drought is the key in drought research. Several years ago, there were many studies indicating that the drought level is fast rising. But many researchers disapproved of this conclusion because those studies used an index that overemphasizes the role of the rising temperature for evapotranspiration. A Nature paper examined this issue and reported that the estimated drought rising rate would be much

The hydrological community has not determined a universally good index to assess drought.

—Qiuhong Tang

smaller if we used another index that more realistically estimates evapotranspiration. This reflects the complexity of drought. The hydrological community has not determined a universally good index to assess drought.

Floods are also very complex. Extreme rainfall is a major trigger, but there are other influencing factors. The impact of climate change is broad. It can raise the intensity of extreme rainfall. It can also raise the sea level, trigger storm surges and change river morphology. For inland cities, extreme rainfall itself can trigger waterlogging; for riverside areas, flood can be result of severe rainfall in the upstream areas; for coastal areas, flood is a mixed result of the sea level, the storm surges and the precipitation. That is to say, climate change can trigger multiple changes, which collectively form hazards.

Another point is that, from a socio-economic perspective, we are particularly interested in the negative consequences of drought and flood. For example, droughts and extreme heat during the growing season may affect crop growth and reduce yields, so we are particularly concerned about the extreme weather disasters in certain seasons and certain agriculture areas. This is important if we want to benefit society with climate science.

Different disciplines do not share the same concept for using the term of ‘attribution’.

—Panmao Zhai

**Zhai:** I agree that different drought indices can give quite different results. Drought is a climate event with relatively large time and space scales, so if we can pinpoint an appropriate drought index, the certainty of drought attribution would be greatly improved.

For climate change impact attribution, there is a problem that different disciplines and different organizations do not share the same concept. For instance, many studies in the hydrologic community simply attribute the impacts to local human activity and climate influence, without considering that human activity also contributes to their ‘climate influence’. They consider only the local human activities, without including the large-scale influences relevant to human activities, such as the global warming trend caused by carbon dioxide emission. In this way, it is not possible to link to projections for the future. There are many other similar concept differences, even between the IPCC Working Groups I and II.

**Tang:** Exactly. The hydrologic community used to treat climate influence as natural impact, and influences of land use/cover change and human water use as human-induced impact. This treatment is arbitrary because human activity contributes to climate change and natural variability can also affect land use/cover change and human water use.

To address this problem, an emerging discipline called ‘Global Change Hydrology’ is trying to build a new framework to scientifically ascertain mechanisms responsible for the changes in the terrestrial water cycle under global change. In this new framework, the water cycle is influenced by a combination of multiple aspects including climate change, land use/cover change, and human water use, and all these aspects are driven by natural and human-induced forces. Human influence on the water cycle includes impacts on several aspects: firstly, climate change caused by human activities; secondly, human-induced land use/cover change, such as the influences of China's afforestation activities on local water cycle and water resources; and thirdly, the influences of human water use such as irrigation and dam building.

**Chen:** I think this is an important issue. We should try to promote this new framework, the success of which may be helpful for the development of China's hydrological research.

## A WARMER AND WETTER NORTHWEST CHINA

**Zhai:** A recent hot topic is that Northwest China, including Xinjiang and part of Tibet is becoming warmer and wetter. Is this trend attributable to human activity and global warming? How long and to what extent will this trend last? These are questions worth answering. The warming and wetting may be beneficial in some sense, offering economic development opportunities for the western and plateau regions. So the related research has both science and social significance.

**Sun:** This is an issue worth further studying and it is also a complex systematic issue involving temperature, water cycle, atmospheric circulation and the interactions among these factors. Our effort to recognize the contributions of diverse factors is just beginning.

## EVOLVING DIRECTIONS OF CLIMATE MODELS

**Tang:** I noticed that many event attribution works followed similar methodology. They do numerical model simulations with or without the influence of external anthropogenic and natural forcing in the system. If the simulated occurrence probability of a certain extreme event with anthropogenic forcing in the model is significantly higher than the result without such forcing, rising from ‘impossible’ to possible, they conclude that human influence has changed the probability of such extreme event. Many works on human influence on precipitation, heat waves and other extreme events were done in this way. Because modelling climate extremes is inherently uncertain, I doubt that in this way we can tune the model so that for many extreme events, we tend to get positive results and attribute them to human activities. I think modelling uncertainty is critical in event attribution.

**Sun:** This method was first proposed by Peter Stott of the UK Met Office and his colleagues in a 2003 *Nature* paper, and is now widely used. A key prerequisite for this method to work is that the climate model has good performance in reproducing these extreme events and the related physical processes. If this prerequisite not met, then the caveat in the calculated results will need to be understood and carefully documented to avoid mis-interpretation. On the other hand, response of extreme events to human influence may differ, depending on the types of events, their space and time scales and physical processes involved. It is generally easier to identify anthropogenic influence at larger scales because of smaller variability and thus larger signal-noise ratio at these scales. It is important to consider all caveats when interpreting results of attribution works.

**Yang:** You mentioned that we have to assume that the models are realistic. But in my study, I noticed that the models are usually not good enough to simulate historical extremes. There are two major problems currently: the resolution is not fine enough to resolve the extreme and the sub-grid parameterization is not that effective and short of scale aware. This leads to some uncertainties in the attribution and projection research.

For example, in infrastructure construction, for disaster reduction and prevention, we need to set an absolute threshold of extreme precipitation, which means setting how much precipitation the building should be able to bear. But the current models are not good at absolute intensity simulation and it is difficult to catch extreme rainfalls heavier than 100 mm. Even though we can tune the model and successfully catch the total amount of extreme rainfall, the physical details in the results are very likely to be wrong. For example, two models give a similar total rainfall amount, but if we look at the vertical profile of atmospheric temperature and moisture, or the vertical distribution of clouds given by the two models, they can be quite different, which could cause large differences for climate feedback between them. Given the limitations of models, trustable projections are still challenging.

There are two major problems currently: the resolution is not fine enough to resolve the extreme and the sub-grid parameterization is not that effective and short of scale aware.

—Jing Yang

**Chen:** You talked about the problems of the models. They are very meaningful and I think the problems can be summarized in three points: resolution, sub-grid parameterization, and the ability to represent physical processes.

Among these points, using downscaling methods to increase the space resolution of models is relevant to the downstream applications. Only when we are able to increase the resolution and deal with a region as small as a city, are we able to guide the industries and city constructions with climate science.

**Zhou:** For downscaling, the Coordinated Regional Climate Downscaling Experiment (CORDEX) under the World Climate Research Programme (WCRP) plays an important role by acting as a bridge connecting global scale, regional scale and local scale. I am confident with the future of regional scale. With the ever-increasing resolution, in particular the quick development of convection permitting models in recent years, our definition for regional scale has downscaled from the 50-km scale to the kilometer scale, and will definitely be further refined.

In Sweden, research organizations and private enterprises are tightly connected.

—Deliang Chen

## SERVICE TO SOCIETY

**Zhou:** Currently, climate change research and social needs are separated in China. Downscaling may become a connecting bridge between them. To create climate research based societal service products, scientists need to cooperate with policymakers

and other stakeholders. We cannot fulfill their demands without understanding their needs.

On the other hand, in China, social sciences and natural sciences are also separated. They use separated funding and language systems. Social scientists and natural scientists seldom communicate with each other and their limited communications are hardly smooth. This is also a barrier for application of climate change research. Future Earth is an international research program, which aims to provide critical knowledge to face the challenges posed by global environmental change. Future Earth introduced into its projects a new concept of research approach, which highlights ‘co-design, co-produce and co-deliver’. This new approach involves stakeholders from the very beginning and aims to fill the gap between science and its supposed end-users.

**Chen:** That is right. In Sweden, research organizations and private enterprises are tightly connected. Researchers offer training and guidance to the enterprises, and many enterprise managers are invited to participate in research funding review and research proposal drafting. These communications set a solid foundation for climate science to serve society.

**Zhou:** CSSP-China is a scientific research project that is building a basis for services to support climate and weather-resilient economic development and social welfare through strong, strategic partnerships harnessing UK scientific expertise. It is supported by the Newton Fund and the Department for Business, Energy & Industrial Strategy (BEIS) UK-China Research Innovation Partnership Fund. Through CSSP-China, strong partnerships between the UK Met Office, the China Meteorological Administration (CMA), the Institute of Atmospheric Physics (IAP) at the Chinese Academy of Sciences, and other key institutes within China and the UK has been developed. The Fund also supports Chinese and British scientists to visit institutes in each other's country.

Before this program, East Asian climate researchers were generally Chinese, Japanese or Korean. This program broke that structure. Now, British researchers are interested in East Asian climate and they have brought a number of advanced research methods and ideas to this field. On the other hand, this program also aims to promote application of basic science in the fields of climate prediction and climate projection, and to tighten the connections between scientists and diverse industries such as agriculture, forestry and hydrology. CSSP-China is already changing the thinking mode of many Chinese scientists.

**Tang:** I have visited the agency responsible for managing flood risk in UK with the support of the Newton Advanced Fellowship. During this visit, I noticed that British insurance industry is tightly connected with the climate change community. The insurance companies offer insurance services for construction of various infrastructures based on assessments of possible economic losses caused by potential natural hazards. This practice is still being developed in China. Currently, China's main disaster relief policy is post-disaster government aid. It may face increasing funding pressure as climate risk increases. A national flood insurance system or climate risk insurance system may be needed and climate science could contribute to the risk management efforts.

**Zhou:** Climate risk assessment is necessary for many construction projects, including infrastructure construction – for example the drainage pipes of a city should be able to withstand a rainstorm once in how many years? In city planning of coastal cities, how high a breakwater should we build for Shanghai and Hong Kong? – as well as many other conditions.

Another example is construction of airports. As the temperature rises, the lifting force the atmosphere can provide to the aircraft drops. If the runway is not built long enough, heavy aircrafts would be unable to take off. There have already been severe flight cancellation events caused by extreme high temperatures in the US. So, quantitative assessment of the climate factors within airport construction has become regular in many countries, but it seems not yet in China. The Paris Agreement set a goal to keep global warming well below 2°C and pursue efforts to limit it to 1.5°C. A recent study found that the half-degree additional warming will lead to a shift toward higher extreme temperature in five Chinese sites: Beijing, Shanghai, Kunming, Lasa and Urumqi. For both 1.5° and 2.0°C scenarios, the number of weight-restriction days will increase significantly at three airports: Beijing, Shanghai, and Lasa.

Climate risk assessment is necessary for many construction projects.

—Tianjun Zhou

**Sun:** Actually, such kinds of assessment also exist in China, but are often done in the provincial institutes and they are more related to the local climate service.

**Yan:** I think it is necessary to call for governmental policies to demand mandatory climate change risk assessment in major infrastructure and construction projects. In that way, we can improve the professionalism and realize the value of the assessments.

**Yang:** Besides the preventive risk assessment, we also lack disaster-related data in China. In August 2019, typhoon Lekima hit Zhejiang province in China. The rainstorm directly submerged a village, causing more than 20 deaths. Scientists have investigated that village and found that this place itself has very high risk of natural disasters and large vulnerability, indicating that it is actually not suitable for human settlement.

Beginning in April 2019, the Ministry of Emergency Management of China started a national disaster risk survey. This survey aims to get accurate historical data for each county of China. Hopefully, these data will provide a solid foundation for future scientific research and disaster prevention and reduction works.

**Yan:** We lack necessary data for disaster risk assessments. For the meteorological and hydrological studies, the issue is that sometimes the data exist but are not open. It will be beneficial if in the future, meteorological and hydrological data as well as those of many other fields could be freely open to researchers and the public, to the extent feasible.

## SUMMARY: VISION AND OUTLOOK FOR THE FUTURE CLIMATE CHANGE RESEARCH

**Chen:** Climate system is a non-linear system. Its behavior is mainly decided by two factors: external forcings and internal feedbacks. What climate change research wants to do is to reliably project future scenarios under different external forcings, to inform our society about consequences of different human choices, such as different levels of carbon dioxide emission. Among these different future scenarios, some are relatively ‘good’ and some are ‘not so good’. We can then choose the behaviors that can lead our society to the better futures. In the climate change field, we often talk about ‘resilience’. But our aim should not be to spring back to what it was, but to move forward to a better future.

